# Estimation of PM2.5 Concentrations in China Using a Spatial Back Propagation Neural Network

**DOI:** 10.1038/s41598-019-50177-1

**Published:** 2019-09-24

**Authors:** Weilin Wang, Suli Zhao, Limin Jiao, Michael Taylor, Boen Zhang, Gang Xu, Haobo Hou

**Affiliations:** 1 School of Resource and Environmental Sciences, Wuhan University, 129 Luoyu Road, Wuhan, 430079 China; 2 Key Laboratory of Geographic Information System, Ministry of Education, Wuhan University, 129 Luoyu Road, Wuhan, 430079 China; 3 Department of Meteorology, University of Reading, Reading, RG6 6BB UK

**Keywords:** Atmospheric dynamics, Atmospheric dynamics, Atmospheric dynamics, Atmospheric dynamics, Environmental impact

## Abstract

Methods for estimating the spatial distribution of PM_2.5_ concentrations have been developed but have not yet been able to effectively include spatial correlation. We report on the development of a spatial back-propagation neural network (S-BPNN) model designed specifically to make such correlations implicit by incorporating a spatial lag variable (SLV) as a virtual input variable. The S-BPNN fits the nonlinear relationship between ground-based air quality monitoring station measurements of PM_2.5_, satellite observations of aerosol optical depth, meteorological synoptic conditions data and emissions data that include auxiliary geographical parameters such as land use, normalized difference vegetation index, elevation, and population density. We trained and validated the S-BPNN for both yearly and seasonal mean PM_2.5_ concentrations. In addition, principal components analysis was employed to reduce the dimensionality of the data and a grid of neural network models was run to optimize the model design. The S-BPNN was cross-validated against an analogous but SLV-free BPNN model using the coefficient of determination (R^2^) and root mean squared error (RMSE) as statistical measures of goodness of fit. The inclusion of the SLV led to demonstrably superior performance of the S-BPNN over the BPNN with R^2^ values increasing from 0.80 to 0.89 and with the RMSE decreasing from 8.1 to 5.8 μg/m^3^. The yearly mean PM_2.5_ concentration in China during the study period was found to be 41.8 μg/m^3^ and the model estimated spatial distribution was found to exceed Level 2 of the China Ambient Air Quality Standards (CAAQS) enacted in 2012 (>35 μg/m^3^) in more than 70% of the Chinese territory. The inclusion of spatial correlation upgrades the performance of conventional BPNN models and provides a more accurate estimation of PM_2.5_ concentrations for air quality monitoring.

## Introduction

Long-term exposure to ambient fine particulate matter (PM) is associated with adverse human health conditions. PM_2.5_ particles, with an aerodynamic diameter <2.5 μm, can be inhaled into the nasal passages and can carry toxic substances that are harmful to human health^1,2^. Studies have shown that long-term exposure to high PM_2.5_ concentrations can have serious impacts on human organs such as the liver, lungs and be responsible for the development of cardiovascular diseases^3–6^. Therefore real time monitoring of PM_2.5_ concentrations is extremely important for preventing pollution-related health issues as well as for the formulation of effective environmental protection measures.

Since 2013, many PM_2.5_ monitoring stations across China have been established to measure air quality data. However, due to their sparse and uneven distribution, with most being located near cities, limitations still exist for effective and representative *in situ* monitoring of PM_2.5_ concentrations at the regional scale^7–9^. Monitoring capability can be increased by fusing satellite remote sensing data such as the aerosol optical depth (AOD) with PM_2.5_ measurements and help in the construction of space-time models of PM_2.5_ concentrations within or across regions. In addition, auxiliary datasets such as meteorological data, source emissions data, land use data, topographic data and socio-economic data, can also been used to reinforce the relationship between PM_2.5_ concentrations and various observed variables^7,9,10^.

Existing models for predicting PM_2.5_ concentrations can be classified into two categories: deterministic models and statistical models^9,11^. Deterministic models, including large-scale air quality simulations^12–14^, model physical processes such as emission, dispersion, transformation, and diffusion, as well as the chemical reactions occurring in polluted air^11,15,16^. However, since most models require sophisticated prior knowledge of pollutant diffusion states and chemical reaction pathways, deterministic PM_2.5_ concentration estimation is complex and computationally expensive^17^. Statistical models, while not necessarily simpler in design, are however able to achieve an almost equivalent level of PM_2.5_ concentration prediction accuracy^18^ and due to their greater speed, have been extensively developed and deployed for monitoring purposes. Linear statistical models including simple linear regression models^19^, multiple linear regression (MLR) models^20^, empirical models^21,22^, and geo-weighted regression models^8,10^, have been able to obtain satisfactory results. However, the functional relationship between the PM_2.5_ concentration and explanatory variables is nonlinear. As a result, many nonlinear statistical models have been used to estimate PM_2.5_ concentrations including support vector regression, generalized additive models^8^, artificial neural network (ANN) models^9,11,18,23,24^ and more recently, deep learning methods^9,11,18,23,24^. With improvements in computational capacity, models have also gradually incorporated more exogenous variables such as meteorological factors (e.g., relative humidity, temperature, and wind speed), land use factors, topographic data, source emissions data and socio-economic data^9,11,18,23,24^.

Despite all these significant advances, most models have ignored the influence of the geographical distance between PM_2.5_ monitoring stations as well as Tobler’s First Law of Geography^25^ - that everything is related to everything else but that nearby things are more related than distant things. Furthermore, various studies have demonstrated that the distribution of PM_2.5_ concentrations shows significant spatial auto-correlation. In this study, we aim to exploit this additional information by developing a spatial back propagation neural network (S-BPNN) that can improve the accuracy of PM_2.5_ concentration estimation by explicitly including a spatial lag variable (SLV). The performance of the S-BPNN model is compared with that of a conventional back propagation neural network (BPNN) that does not include the SLV. We then use the S-BPNN to map the yearly and seasonal mean distribution of PM_2.5_ concentrations across China for the study period, and assess exceedances.

## Data and Methods

### Data fusion

In order to construct a S-BPNN multivariate model, ground-level PM_2.5_ concentration measurements were fused with satellite aerosol optical depth data, meteorological synoptic conditions data and source emissions data at 1280 monitoring sites in China, to form a large and spatially-diverse sample dataset of seasonal and yearly mean values. Table S1 presents the sources, units and spatial scales of each variable. Arcpy in ESRI’s ArcGIS was used to perform spatial interpolation for meteorological data as part of the preparation of the sample data.

#### Ground-level PM_2.5_ measurements

**Figure 1:**
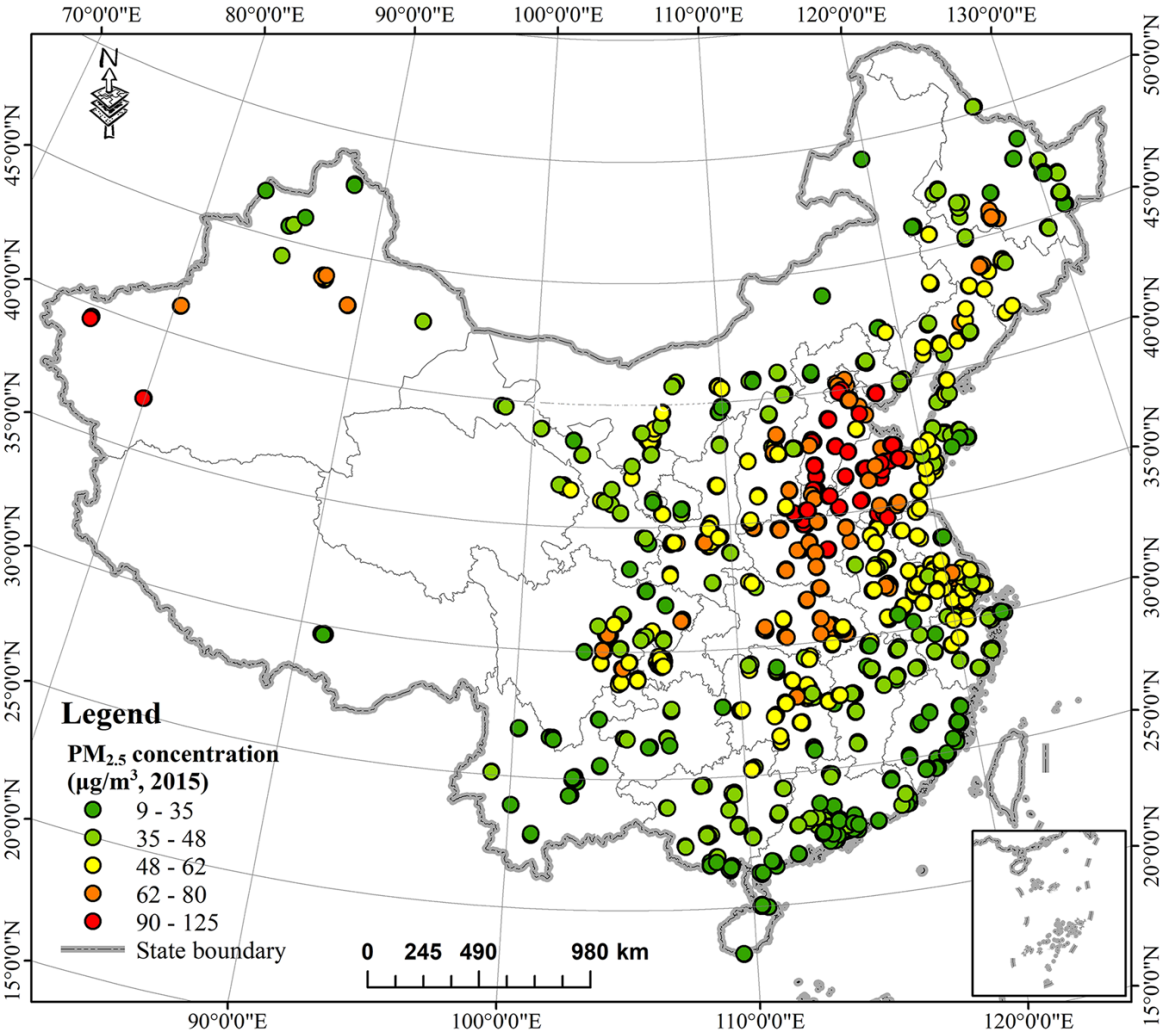
ArcGIS map of the distribution of ground-level monitoring sites in China, 2015.

Hourly PM_2.5_ concentrations at 1280 stationary sites in 190 cities from 2015-01-01 to 2015-12-31 were collected from the official database of the China National Environmental Monitoring Centre (CNEMC: http://www.cnemc.cn/en/). PM_2.5_ concentrations were measured via the tapered element oscillating microbalance method (TEOM) and then averaged at each site to produce time series of daily mean PM_2.5_. Seasonal (spring: MAM, summer: JJA, autumn: SON, winter: DJF) and yearly mean PM_2.5_ concentrations were then also calculated from the daily mean PM_2.5_ for all monitoring stations. Figure 1 shows the spatial distribution of these PM_2.5_ monitoring sites in China and the yearly mean value for 2015.

#### MODIS AOD satellite data

The AOD at 550 nm from the moderate resolution imaging spectraradiometer (MODIS) has been found to weakly correlate with PM_2.5_ concentrations^11,26,27^. Nonetheless, this is an important explanatory variable used to drive satellite remote sensing models of PM. We obtained AOD data from the MODIS Terra and Aqua Collection 6.1 via the NASA Level-1 and Atmospheric Archive and Distribution System (https://ladsweb.modaps.eosdis.nasa.gov). The AOD data has a maximum spatial resolution of 10 km and covers the study period from 2015-01-01 to 2015-12-31. The 10 km AOD products were retrieved using the Dark Target (DT) algorithm and the MODIS Conversion Toolkit (MCTK). For each grid cell, where Terra MODIS AOD (MOD04) data was available, we estimated missing Aqua MODIS AOD (MYD04) data by linear interpolation to extract values at the centre of the pixel. The same estimation procedure was used to for MOD04 when only MYD04 was available. MOD04 and MYD04 data was averaged where both products were available. Daily AOD products were then averaged to produce seasonal and yearly AOD values.

#### Synoptic conditions data

Meteorological data was then obtained from the Meteorological Data Sharing Service System in China (http://data.cma.cn/en) and includes wind speed (WS, m/s), relative humidity (RH, %), surface pressure (PRS, Pa), temperature (TEM, °C), precipitation (PRE, mm), and sunshine duration (SSD, h). There are 839 meteorological monitoring stations providing a total of 306,235 records during the study period. To obtain seasonal means and yearly means, we averaged the seasonal and yearly mean WS, RH, PRE, TEM, PRE, and SSD values calculated from the daily meteorological data across the monitoring network. Yearly mean distribution maps of each meteorological variable in the study area were then spatially interpolated with Arcpy in ESRI’s ArcGIS software by calculating the seasonal and yearly mean meteorological conditions at the monitoring stations with the inverse distance weighted (IDW) using a grid size of 10 km × 10 km. Values are extracted from the centre of 10 km grid.

#### PM_2.5_ emissions data

Land use is a major contributor to the source apportionment of PM_2.5_ pollution^7^. Land use data for 2015 having 30 metre resolution was obtained from the Geographical Information Monitoring Cloud Platform (http://www.dsac.cn/) and categorised into built-up areas, arable land, forest, water bodies, and bare land. We also downloaded NDVI and population density data having spatial resolution 1 km × 1 km from the Resource and Environment Data Cloud Platform (http://www.resdc.cn/) and LandScan (https://web.ornl.gov/sci/landscan/) respectively. In both cases, we extracted the pixel value closest to each PM_2.5_ monitoring station. To account for the contribution of traffic emission pollution sources to the PM_2.5_ concentration^7^, main roads within 10 km of the PM_2.5_ monitoring sites were included using road network data downloaded from OpenStreetMap (www.openstreetmap.org/). To account for industrial sources of pollution, we considered the number and distribution of state monitoring enterprises for exhaust gas emissions as being indicative of the number and distribution of industrial pollution sources. The basic information (including addresses) of the exhaust gas monitoring enterprises in 2015 (totaling 3206) was obtained from the Ministry of Ecology and Environment of the People’s Republic of China (http://www.mee.gov.cn/). The Baidu Geocoding API (http://lbsyun.baidu.com/index.php?title=webapi/guide/webservice-geocoding) was then used to obtain the geographical location (latitude and longitude) of each enterprise. The number of emission enterprises within a radius of 10 km of the PM_2.5_ monitoring stations was used as a measure of industrial emissions. Finally, digital elevation model (DEM) data was derived from the Geospatial Data Cloud (http://www.gscloud.cn/) and the pixel value containing or nearest to each PM_2.5_ station was extracted.

Figure S1 presents the histograms and associated median statistics for the set of variables in the fused sample data set spanning the period 2015-01-01 to 2015-12-31 calculated from the 1280 PM_2.5_ ground-based monitoring sites in China. The median value of the PM_2.5_ concentration is 52.0 μg/m^3^. The distributions of AOD at 550 nm, WS, temperature, SSD, NDVI and construction land area exhibit some similarity to the distribution of PM_2.5_ concentrations. In contrast, other variables including RH, pressure, precipitation, population density, road length, DEM base height, and the number of industrial pollution companies have distributions that are either multimodal or power law in nature. While these variables aren’t expected to strongly co-vary with the PM_2.5_ concentration, recent studies have shown that they can nevertheless still have an influence on the spatial distribution of PM_2.5_ concentrations^7,9,18,26^. Consequently, we performed principal components analysis (PCA) on the full set of parameters, retaining those components that account for >98% of the total variance in line with recent approaches^24,28^. As a result, the dimensionality of the was reduced from 15 variables (AOD, latitude, longitude, RH, WS, temperature, pressure, precipitation, NDVI, DEM, population density, number of pollution companies, road length, construction land area plus the SLV calculated from localised PM_2.5_ concentrations described in the next section) to 11 principal components.

### Spatial autoregression

In order to develop a continuous model for the distribution of PM_2.5_ concentrations over China as from local measurements made at air quality network monitoring stations together with regionally-distributed independent variables, we construct a spatial autoregressive (SAR) model. SAR models are a class of statistical models that apply to observations over a continuous spatial domain typically made at local nodes of a network or vertices of a uniform or non-uniform grid. Importantly, they allow the effect of spatial correlation to be included explicitly. SAR extends conventional multiple linear regression by allowing outcomes in one area to be affected by outcomes in nearby areas (i.e. spatial lags on the dependent variable), by covariates from nearby areas (i.e. spatial lags on independent variables), and by spatially autoregressive errors from nearby areas. The general form for a first-order SAR model is given by^29,30^:

1 $$\begin{array}{c}{\boldsymbol{y}}={\bf{X}}\beta +\rho {{\bf{W}}}_{1}{\boldsymbol{y}}+{\boldsymbol{u}}\\ {\boldsymbol{u}}=\lambda {{\bf{W}}}_{2}{\boldsymbol{u}}+{\boldsymbol{\varepsilon }}\\ {\boldsymbol{\varepsilon }}\sim N(0,{\sigma }^{2}{{\boldsymbol{I}}}_{n})\end{array}$$

where ***y*** is a [n × 1] scalar vector of observations of the dependent variable (PM_2.5_ concentrations in our case), **X** is a [n × k] matrix of exogenous variables with [k × 1] regression coefficients β, **W**_1_***y***, is the [n × 1] spatial lag variable (SLV) calculated from the weighted average of nearby PM_2.5_ monitoring sites (see next section) with spatial autoregression parameter ***ρ***, ***u*** is the error term expressed in terms of [n × 1] spatially-lagged errors **W**_1_***u***, with spatial autoregression coefficient **λ** and ***ε*** which is a [n × 1] scalar vector of normally distributed (iid) random errors. **W**_1_ and **W**_2_ are the [n × 1] spatial weights matrices and in geophysical applications, are usually equivalent with the general property that all of their diagonal elements are zero and their rows sum to one. Equation (1) is sufficiently general that it embraces two major classes of spatial statistics models.

For ρ = 0, Eq. (1) reduces to the spatial error model (SEM) whereby the spatial dependence of ***y*** is correlated with spatial autoregression in the errors and measures the influence of errors in the exogenous variables in the local neighbourhood of observations of the dependent variable:

2 $${\boldsymbol{y}}={\bf{X}}\beta +{({\bf{I}}-\lambda {{\bf{W}}}_{2})}^{-1}{\boldsymbol{\varepsilon }}$$

For λ = 0, Eq. (1) reduces to the spatial lag model (SLM) whereby ***y*** is spatially-autocorrelated and models the diffusion of ***y*** over a region:

3 $${\boldsymbol{y}}={({\bf{I}}-\rho \lambda {{\bf{W}}}_{1})}^{-1}{\bf{X}}\beta +{({\bf{I}}-\rho \lambda {{\bf{W}}}_{1})}^{-1}{\boldsymbol{\varepsilon }}$$

The SLM therefore incorporates a spatial multiplier (**I** − ρ**W**_1_)^−1^ called the “Leontief inverse” which connects the dependent variable ***y*** to all exogenous variables *x*_*i*_ in the system at location *I* and not just the error at location *i*. This observed behaviour has been reported in several studies that have shown that the distribution of PM_2.5_ concentrations show significant spatial autocorrelation^11,26^. For a specific grid, the SLV is given by:

4 $$SLV=\frac{\mathop{\sum }\limits_{i=1}^{n}w{s}_{i}P{M}_{2.5,i}}{\mathop{\sum }\limits_{i=1}^{n}w{s}_{i}}$$

where, *n* is the number of nearby PM_2.5_ concentration measurements, *ws*_*i*_ = 1/*ds*_*i*_ is the spatial weight for the *i*^th^ nearby PM_2.5_ concentration and *ds*_*i*_ is its spatial distance. SAR modeling with Arcpy in ESRI’s ArcGIS suggests that n = 3 is optimal for our sample data set. In order to construct an integrated model that not only reflects the local autocorrelation of the PM_2.5_ concentrations but also expresses the nonlinear relationship between PM_2.5_ concentration and independent variables, we explicitly incorporate the SLV as a virtual variable and construct a S-BPNN.

### Spatial back-propagation neural network (S-BPNN)

**Figure 2:**
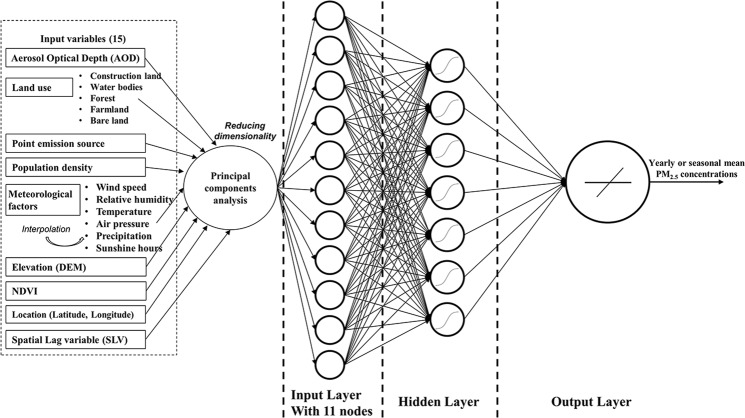
Schematic of the S-BPNN used to estimate PM_2.5_ concentration in China.

Previous studies confirm that ANN models perform more efficiently than MLR models for PM air pollution monitoring and forecasting^9,18,31^ due to their increased capacity for modeling the nonlinear relation between exogenous variables and PM_2.5_ concentrations. A conventional BPNN with three layers (an input layer, a hidden layer and an output layer) was constructed in our study as a control model as it has the desired property that it can act as a universal function approximator (UFA)^32–34^ and provide reliable baseline results. We then investigate the effect of including spatial autoregression by incorporating the SLV as an additional virtual variable in the set of exogenous inputs. We refer to the resulting model as a S-BPNN model of PM_2.5_ concentration. As described in the section on Data Fusion, PCA was applied to the list of 15 exogenous parameters that include the SLV and the resulting 11 principal components, accounting for >98% of the total variance, were used as explanatory variables in the input layer. In accordance with the requirements of a UFA^35^ the hidden layer comprises neurons having a nonlinear activation function. The output layer is a single linear neuron providing the PM_2.5_ concentration. A schematic diagram of the S-BPNN is illustrated in Fig. 2.

### Optimization of the S-BPNN model structure

When optimising the design of feed-forward back-propagation neural networks with supervised learning, training accuracy is expected to increase with the addition more neurons added to a hidden layer. However, this does not then necessarily translate into a corresponding improvement in overall model accuracy when tested on ‘unseen’ validation data. Apart from the obvious increase in training time needed, the problem is over-fitting of the neural network to the training data; resulting in a loss of ability to generalise on new data; significantly and negatively impacting overall model validity and performance^24,32^. Whether a top-down approach to selecting the number of neurons with network weight pruning or a bottom-up convergence approach is adopted, the goal for setting the number of nodes in the hidden layers of feed-forward neural networks is to use as few nodes as necessary to meet the accuracy requirements of the model. Studies have shown that approximate bounds on the numbers of nodes in the hidden layer for most applications range from $2\sqrt{{\rm{n}}}+{\rm{\mu }}$ to2n + 1^9,36^, where n and μ are the numbers of nodes in the input layer and output layer, respectively. With a single output and 11 inputs from PCA, this suggests that the optimal number of nodes in the hidden layer of the S-BPNN required to maximise performance ought to be in the range 7 to 23. Following previous studies^9,24,33^, we applied a sensitivity analysis approach to the neural network architecture by running a grid of models varying the number of nodes in the hidden layer from 1 to 33 and monitoring the model accuracy. Each neuron in the hidden layer had a nonlinear logistic activation function $f(x)=\frac{1}{1+{e}^{-x}}$ and for weight and bias optimisation, MATLAB’s “traingdx” back-propagation algorithm was adopted which performs gradient descent with momentum (0.9) and an adaptive learning rate (0.05) using the mean squared error (MSE) as a cost function.

Figure S2 shows the training and validation goodness of fit statistics for the grid of runs used to optimise the architecture of the S-BPNN. In the evaluation of each run, 10-fold cross-validation was applied to the sample data divided 70%: 30% into a training (model fitting) dataset containing 896 records and a test (validation) dataset containing 384 records. The statistical indicators used to measure the goodness of fit are the coefficient of determination (R^2^), the root-mean-square error (RMSE, μg/m^3^), the mean prediction error (MPE, μg/m^3^), and relative prediction error (RPE, %) which are defined in the Supplementary Information accompanying this paper. As expected, we observed that, as the number of neurons in the hidden layer increases, S-BPNN model performance slightly improves for the training (fitting) data but gradually degrades for the test (validation) data. Over-fitting is observed when the number of neurons > 7 (the point beyond which the R^2^ value continues to degrade from its first local maximum). As such, 7 hidden neurons were adopted as the optimal case. We also investigated the effect of changing the back-propagation training algorithm to the Levenberg-Marquardt algorithm and using the hyperbolic tangent (*tanh*) nonlinear activation function, but neither led to any improvement in the performance.

## Results and Discussion

### Performance of the models

After determining the optimal neural network architecture, we then partitioned the sample dataset comprising N = 1280 records (one for each monitoring station) including yearly and seasonal mean data, and trained and tested the S-BPNN and BPNN. A common framework was used for both models, with the exception that the S-BPNN includes the SLV in the principal components fed as inputs to the network. MATLAB’s Neural Network Toolbox version 6.0 was then used to build and train the S-BPNN and the BPNN. The accuracy of the trained models was calculated using 10-fold cross-validation. During this procedure, the sample data set was randomly divided into 10 parts; 9 of which were used for fitting and 1 for validation each time. The mean accuracy of 10 cross-validation trials for the networks trained on yearly mean data is shown in Table 1.

**Table 1:** Accuracy of the trained S-BPNN and BPNN models calculated with 10-fold cross-validation applied to yearly mean data.

Model	Index	Fitting				Validation			
		R^2^	RMSE	MPE	RPE (%)	R^2^	RMSE	MPE	RPE (%)
**S-BPNN**	Min	0.89	5.80	4.25	11.10%	0.85	5.03.	3.73	9.66%
	Mean	0.89	5.80	4.30	11.14%	0.89	6.03	4.46	11.57%
	Max	0.90	5.80	4.36	11.20%	0.92	7.45	5.17	13.91%
**BPNN**	Min	0.77	7.83	6.07	15.02%	0.65	7.77	6.24	14.87%
	Mean	0.80	8.09	6.27	15.54%	0.75	9.03	6.95	17.36%
	Max	0.81	8.70	6.65	16.61%	0.83	10.16	7.85	19.74%

The units of RMSE and MPE are μg/m^3^.

**Figure 3:**
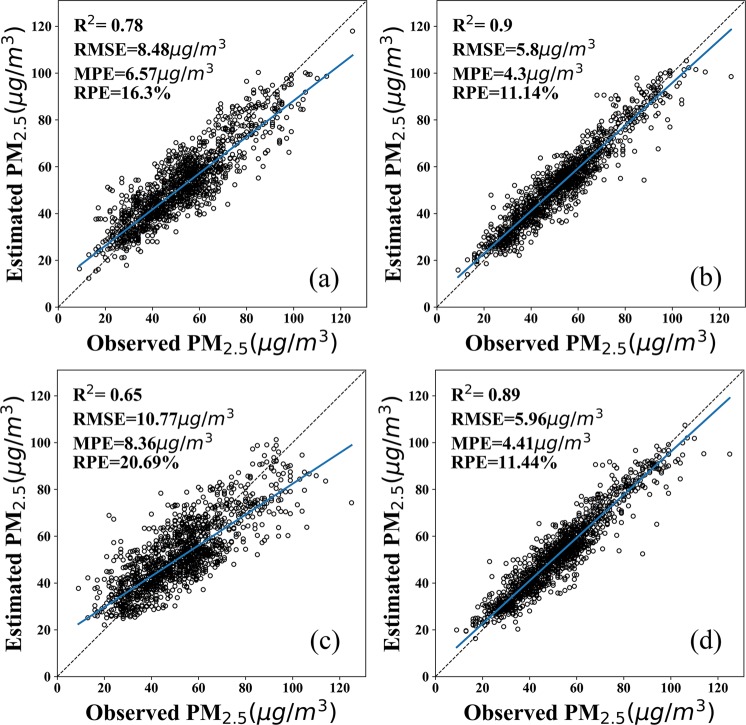
Scatter plots of BPNN and S-BPNN fitting and validation results for yearly mean data. The solid line is the trend line and the dashed line is the 1:1 line as a reference. (**a**) and (**c**) are the BPNN model fitting and 10-fold cross-validation results, respectively. (**b**) and (**d**) are the S-BPNN model fitting and 10-fold cross-validation results, respectively.

Inclusion of the SLV in the S-BPNN leads to an increase in performance with the mean R^2^ of the fitting and validation data increasing from 0.80 and 0.75 respectively for the BPNN to 0.89 for the S-BPNN. A corresponding reduction in all mean error measures is observed. The RMSE of the S-BPNN never exceed 7.45 μg/m^3^. To further evaluate the performance of S-BPNN and BPNN models, scatter plots of the fitting and validation results are shown in Fig. 3.

To assess the performance on the seasonal timescale, the same approach was adopted and applied to seasonal mean datasets to train and evaluate S-BPNN and BPNN models. The mean accuracy of 10 cross-validation trials for the networks trained on the seasonal mean data are presented in Table 2. Some variation in model performance with season is apparent with lowest errors in the summer and largest errors in the winter seasons. This is to be expected for two reasons. Firstly, cloud cover is seasonal and impacts the quality of satellite AOD retrievals. While uncertainty on the model inputs was not explicitly included in the model design in this study, the propagation of uncertainty through spatial nonlinear models warrants future attention. Secondly, the sample data is constructed from daily averaging which leads to variation in the uncertainty of the sample data used to train the models at this timescale. This variation is expected to impact the quality assessment of models as their prediction timescale (yearly → seasonal) approaches the daily timescale. Nevertheless, it is clear that the S-BPNN outperforms the BPNN in all cases and that the incorporation of spatial information is of clear benefit even at shorter modelling timescales.

**Table 2:** Accuracy of the trained S-BPNN and BPNN models calculated with 10-fold cross-validation applied to seasonal mean data.

Season	Model	Index	Fitting				Validation			
			R^2^	RMSE	MPE	RPE (%)	R^2^	RMSE	MPE	RPE (%)
Spring	S-BPNN	min	0.76	8.02	5.56	16.93	0.60	7.54	5.08	15.45
		mean	0.77	8.21	5.63	17.42	0.75	8.65	5.88	18.40
		max	0.78	8.32	5.77	17.72	0.81	10.81	6.64	23.84
	BPNN	min	0.62	9.78	7.11	20.62	0.47	9.64	7.12	19.68
		mean	0.65	10.17	7.50	21.56	0.59	10.87	7.98	23.12
		max	0.67	10.7	7.96	22.78	0.74	12.90	9.34	28.64
Summer	S-BPNN	min	0.72	7.07	4.84	19.43	0.60	6.14	4.35	17.50
		mean	0.73	7.30	4.96	20.11	0.69	7.96	5.33	21.95
		max	0.74	7.52	5.10	20.63	0.77	9.63	5.99	26.99
	BPNN	min	0.56	8.62	6.33	23.55	0.39	8.63	6.34	23.93
		mean	0.59	8.93	6.58	24.61	0.51	9.72	7.19	26.87
		max	0.63	9.19	6.76	25.50	0.60	11.01	7.94	31.22
Autumn	S-BPNN	min	0.70	9.13	6.14	19.12	0.32	7.96	5.75	16.69
		mean	0.71	9.70	6.27	20.35	0.68	10.24	6.63	21.52
		max	0.75	9.98	6.36	20.92	0.80	16.01	8.36	34.24
	BPNN	min	0.60	10.66	7.58	22.31	0.44	9.97	7.78	21.03
		mean	0.63	11.06	7.98	23.22	0.57	11.83	8.59	24.82
		max	0.66	11.62	8.41	24.37	0.70	14.8	9.50	30.94
Winter	S-BPNN	min	0.80	13.18	8.94	16.68	0.74	11.64	8.39	14.66
		mean	0.82	13.58	9.09	17.19	0.79	14.39	9.67	18.21
		max	0.83	14.08	9.31	17.74	0.88	16.51	10.76	20.87
	BPNN	min	0.71	16.21	11.96	20.54	0.61	15.57	12.52	19.45
		mean	0.72	16.74	12.37	21.19	0.68	17.83	13.27	22.59
		max	0.74	17.2	12.82	21.80	0.76	18.97	15.09	25.24

The units of RMSE and MPE are μg/m^3^.

### Spatial distribution of PM_2.5_ concentration

**Figure 4:**
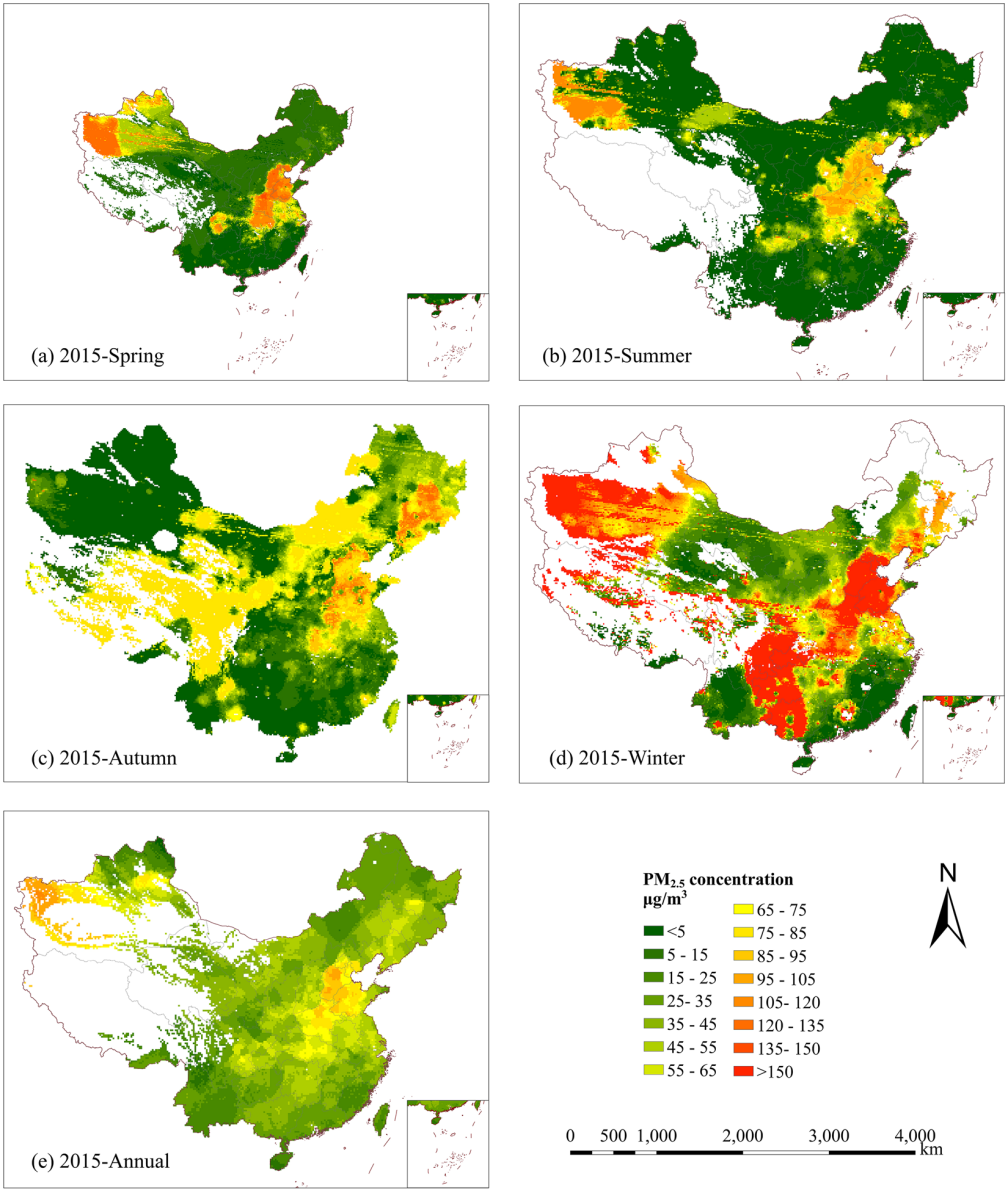
Spatial distributions of seasonal and annual estimated PM_2.5_ concentrations (μg/m^3^) in China, 2015: (**a**) spring, (**b**) summer, (**c**) autumn, (**d**) winter and (**e**) annual (Jan. 2015 to Dec. 2015). The white regions indicate missing data. Maps were made using ArcGIS software.

In Fig. 4, the S-BPNN model was used to map the seasonal and yearly mean distribution of PM_2.5_ concentrations in China at a spatial resolution of 10 km. The distributions of seasonal and annual PM_2.5_ concentrations have considerable spatial heterogeneity and spatial aggregation. This has two major impacts. Firstly, the sparse monitoring of large expanses of south-western China leads to significant gaps in model input data in this region. Secondly, strong clustering of monitoring stations in regions of high urbanisation means that spatial data in these regions is likely to be more representative. The S-BPNN modeled yearly mean PM_2.5_ concentration for China in 2015 was found to be 41.76 μg/m^3^ and reflects well the value of 52.0 μg/m^3^ calculated at the nodes of the air quality monitoring network. Importantly, the interpolative power of the S-BPNN model at interstitial locations allows for a moderate resolution assessment of national exceedances nationwide. We find that more than 70% of Chinese territory exceeds Level 2 of the Ambient Air Quality Standards (CAAQS) having a yearly mean concentration > 35 μg/m^3^.

Overall, the levels of PM_2.5_ concentrations are higher in the northern regions than in the southern regions. Heavily polluted regions are located in the North China Plain, especially Beijing-Tianjin-Hebei (BTH), and south-western Xinjiang, where the highest annual PM_2.5_ concentration reached 108 μg/m^3^. However, the causes for such high levels of PM_2.5_ are different in these locations. Pollution in the North China Plain’s is caused mainly by industrial emissions and is exacerbated by stagnant weather, with a weak wind and a relatively low boundary layer height reducing the dispersion, transformation and diffusion of atmospheric gases and chemical reactions^26^. South-western Xinjiang’s pollution is due to desert dust particles which make a significant contribution to the accumulation of PM_2.5_ concentrations^7^. While lower level regions of PM_2.5_ concentrations are found in the south provinces (e.g., Hainan, Guangdong, Fujian and Yunnan), these regions benefit from low levels of industrial source emissions and favourable meteorological conditions for gas dispersion and chemical reaction in the atmosphere. While it is immediately apparent that winter is the most polluted season with high levels of PM_2.5_ concentrations and summer is the cleanest with the lowest levels, some regions also exhibit high levels of PM_2.5_ concentrations in the spring especially in the North China Plain and over Northwest China. PM_2.5_ pollution is mitigated to some extent in the autumn months.

## Conclusions

Faced with the complexity involved in modelling PM_2.5_ concentrations deterministically, neural network-driven statistical models like BPNNs have been developed with demonstrable advantages for the estimation and mapping of PM_2.5_ concentrations and other particulate matter components of air pollution^9,24,37–40^. By incorporating spatial correlation information using a SLV in an S-BPNN model design, we were able to improve the accuracy and performance of a BPNN trained to retrieve PM_2.5_ concentrations at 10 km resolution from satellite AOD, meteorological data, land use data, source emission data and related geographical data. The exogenous variables used encompass not only static features that impact PM_2.5_ concentrations, but also the dynamic processes at work at the local and regional scale.

Cross-validation results suggest that the S-BPNN model outperforms conventional BPNN models and provides accurate estimates of yearly mean PM_2.5_ concentrations and exceedances for China to a precision of RMSE < 7.45 μg/m^3^. Similarly reliable estimates were also obtained for seasonal means over China, recovering understood patterns in the geographical distribution of pollution sources across the country both in terms of geography and also in terms of synoptic conditions. Sensitivity analysis applied to neural network design using a grid of runs in combination with 10-fold cross-validation enabled model performance to be optimised in a systematic way, and ensured that the models produced are robust and reproducible.

Despite the satisfactory performance of the S-BPNN model, the SLV only accounts for the spatial distance between PM_2.5_ monitoring stations. It is possible that a further increase in performance can be achieved by extending the SAR model to include also the spatial lag of covariates as well as spatially autoregressive errors both within and between spatial domains. In particular, it is expected that the inclusion of and propagation of uncertainty information on the variates will to help models capture higher frequency variability PM concentrations. It is also not yet clear what the balance is between increased SAR model complexity and nonlinearity in terms of the efficiency and performance of statistical models of PM_2.5_ concentrations, i.e. whether or not a linear framework could be adequate. In a future study we will adapt the SLM to MLR models of PM and consider other approaches such as deep learning to help identify spatial features and model trends in the data. For this, we will exploit the availability of high-resolution satellite AOD products to map at even higher resolution greater detail, to estimate the distribution of PM_2.5_ concentrations.

## Supplementary information

Estimation of PM2.5 Concentrations in China Using a Spatial Back Propagation Neural Network

## Data Availability

The research data sets used in this work are available upon request from Limin Jiao (lmjiao@whu.edu.cn). Access to monitoring data is permitted subject to the consent of the respective observatories owning the source instruments, and according to their internal policies for data administration. Please refer the author list for contact details.
