# Supplementary material for: Estimation of PM2.5 Concentrations in China Using a Spatial Back Propagation Neural Network
Source: Sci Rep. 2019 Sep 24;9:13788. doi: 10.1038/s41598-019-50177-1 (PMC6760143; doi:10.1038/s41598-019-50177-1)
Supplement: Supplementary file 1 — Estimation of PM2.5 Concentrations in China Using a Spatial Back Propagation Neural Network [file 41598_2019_50177_MOESM1_ESM.docx]

Supplementary Information

W. Wang, S. Zhao, L. Jiao, M. Taylor, B. Zhang, G. Xu, H. Hou. Estimation of PM2.5 Concentrations in China Using a Spatial Back Propagation Neural Network.

Table of Contents

[Supplementary Information 1](#_Toc6839162)

[Glossary of terms and abbreviations 1](#_Toc6839163)

[Supplementary Statistics 2](#_Toc6839164)

[Supplementary Table 2](#_Toc6839165)

[Supplementary Figures 3](#_Toc6839166)

# Glossary of terms and abbreviations

ANN Artifical Neural Network

AOD Aerosol Optical Depth

BPNN Back-Propagation Neural Network

CAAQS China Ambient Air Quality Standards

CLA Construction Land Area

CNEMC China National Environmental Monitoring Centre

DEM Digital Elevation Model

IQR Inter-Quartile Range

MLR Multiple Linear Regression

MODIS Moderate Resolution Imaging Spectroradiometer

MPE Mean Prediction Error

NDVI Normalized Difference Vegetation Index

PCA Principal Components Analysis

PM Particular Matter

R^2^ Coefficient of Determination

RH Relative Humidity

RMSE Root Mean Squared Error

RPE Relative Prediction Error

SAR Spatial Autoregression

S-BPNN Spatial Back-Propagation Neural Network

SEM Spatial Error Model

SLM Spatial Lag Model

SLV Spatial Lag Variable

SSD Sunshine Duration

TEOM Tapered Element Oscillating Microbalance

UFA Universal Function Approximator

WS Wind Speed

# Supplementary Statistics

The statistical indicators used to measure model performance are based on differences and include correlation coefficient (R^2^), the root-mean-square error (RMSE, μg/m^3^), the mean prediction error (MPE, μg/m^3^), and relative prediction error (RPE, %) defined as follows:

where $n$ is the total number of data records, and ${PM}_{o}, {PM}_{e}$ are the observed PM_2.5_ concentration and model-estimated PM_2.5_ concentrations, respectively. $\bar{{PM}_{o}}, \bar{{PM}_{e}}$ are the mean PM_2.5_ concentrations of observation and model-estimated, respectively.

# Supplementary Table

**Table S1**. Data sources, spatial scales and units for each variable in the sample data set.

| Category | Variable | Units | Spatial scale | Data source |
| --- | --- | --- | --- | --- |
| Measurements | PM_2.5_ | μg/m^3^ | N/A | Official database of the China Environmental Monitoring Center (CEMC): <http://106.37.208.233:20035/> |
| Satellite data | AOD (550nm) | N/A | 10 km | Atmospheric Archive and Distribution System (LAADS Web): http://ladsweb.nascom.nasa.gov/data/search.html |
| Synoptic conditions | WS | m/s | N/A | Atmospheric Archive and Distribution System (LAADS Web (http://data.cma.cn/) |
|  | RH | % | N/A |  |
|  | Pressure | Pa | N/A |  |
|  | Temperature | ℃ | N/A |  |
|  | Precipitation | mm | N/A |  |
|  | SSD | h | N/A |  |
| PM2.5 source emission data | Construction land area | km^2^ | 10 km | GIMCP: <http://www.dsac.cn/> |
|  | Road length | km | 10 km | OpenStreetMap: [www.openstreetmap.org/](http://www.openstreetmap.org/) |
|  | NDVI | km | 1km | Resource and Environment Data Cloud Platform: http://www.resdc.cn/ |
|  | Population density | people | 1 km | LandScan: <https://web.ornl.gov/sci/landscan/> |
|  | Pollution sources |  | N/A | MEP website: <http://www.mep.gov.cn/> |
|  | DEM base height | m | 30 m | Geospatial Data Cloud: <http://www.gscloud.cn/> |

# Supplementary Figures


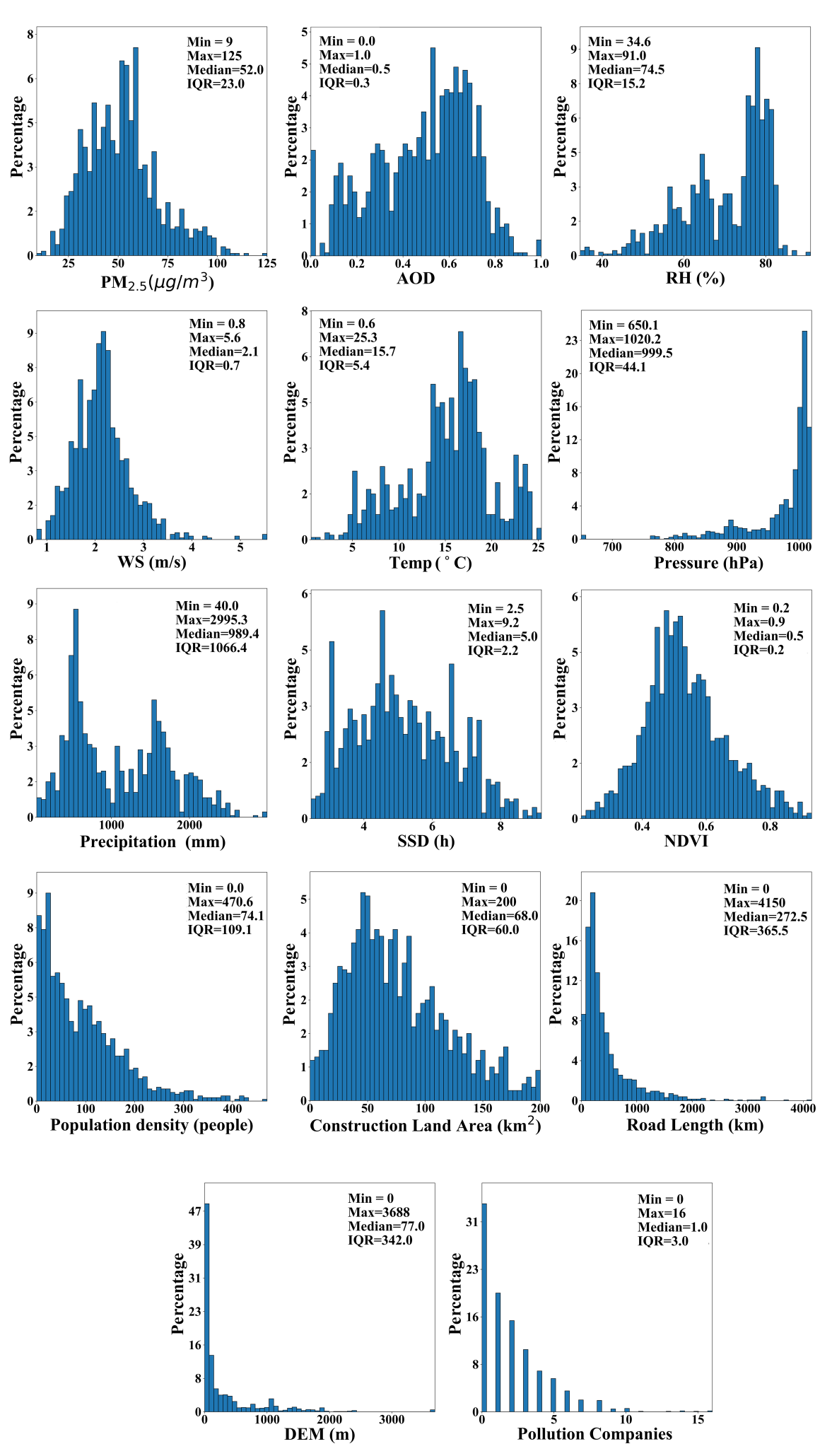


**Figure S1.** Histogram and median statistics of the candidate explanatory variables used in construction of the S-BPNN model. The sample data is drawn from N = 1280 monitoring sites across China.


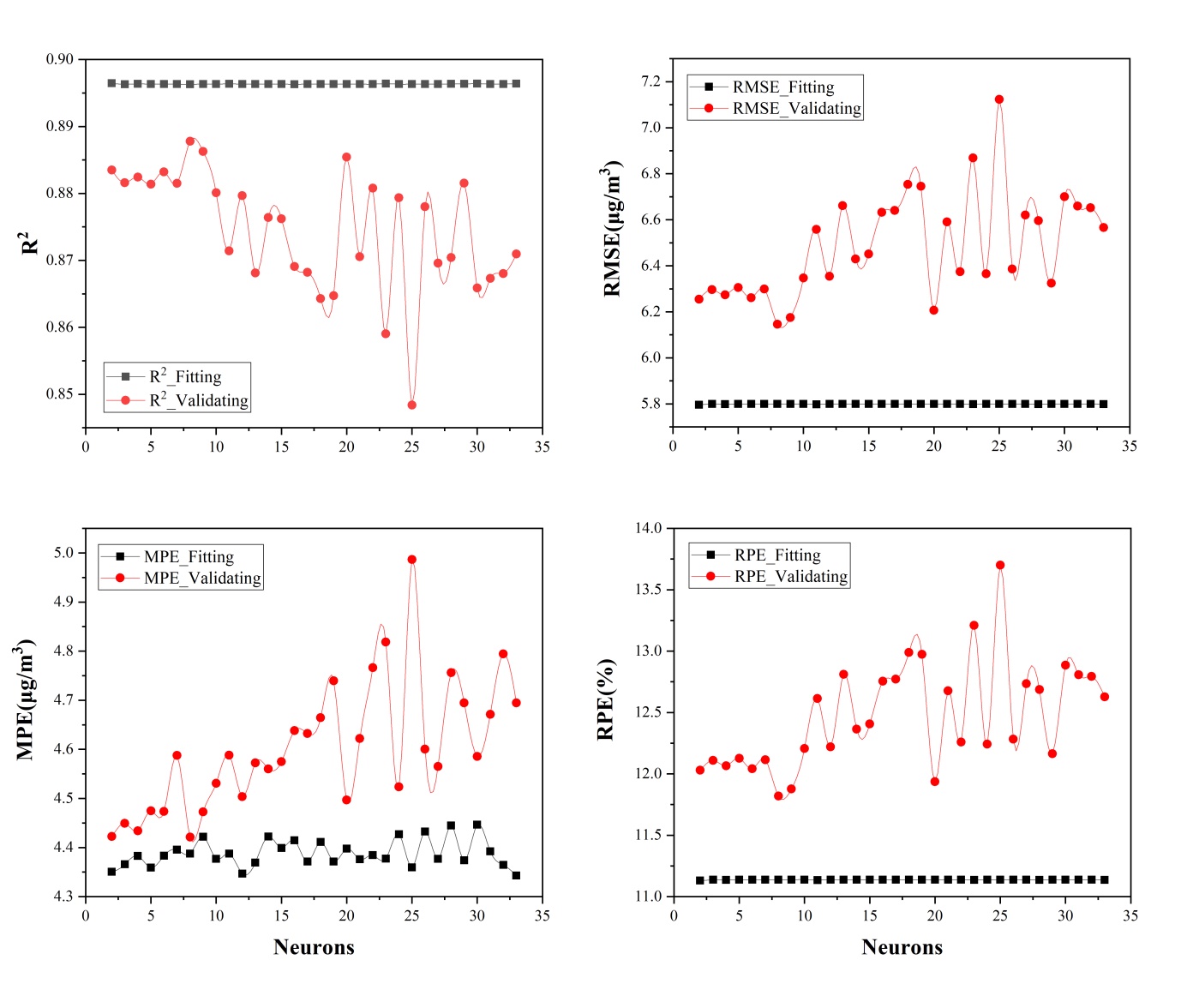


**Figure S2.** S-BPNN model performance as a function of the number of neurons in the hidden layer.
